# Locked volar plating for complex distal radius fractures: Patient reported outcomes and satisfaction

**DOI:** 10.1186/1749-799X-5-51

**Published:** 2010-08-05

**Authors:** RE Anakwe, LAK Khan, RE Cook, JE McEachan

**Affiliations:** 1 The Hand Service, Department of Trauma & Orthopaedic Surgery Queen Margaret Hospital, Whitefield Road, Dunfermline, KY12 0SU, UK

## Abstract

**Background:**

Distal radius fractures are common. The increasing prevalence of osteoporosis contributes to frequently complex articular injuries sustained even after low energy falls. The best method of treating complex type C distal radius fractures is debated. Locked volar plating and external fixation are both widely used with good reported results. Measures of success are traditionally based on technical measurements or the perception of the surgeon. Patient reported measures of outcome are increasingly recognised as important markers of surgical success. We report our experience using locked volar plating for complex type C distal radius fractures as well as patient reported measures of success and satisfaction.

**Methods:**

Over a 12 month period we treated 21 patients with type C distal radius fractures using locked volar plating. These patients were followed up for at least 12 months and the outcome was assessed using clinical examination, grip strength measurements, radiographs and Patient Rated Wrist Evaluation (PRWE) scoring.

**Results:**

The 21 patients studied had an average age of 48 years. There were 8 men and 13 women. All of the fractures had united by 3 months. There were no cases of wound infection or tendon injury/irritation. Patients reported low pain scores, good patient rated wrist evaluation scores and high levels of satisfaction.

**Conclusions:**

Locked volar plating for complex distal radius fractures produces good results when assessed using patient reported measures of outcome. Further work should address whether locked volar plating offers superior outcomes and patient satisfaction compared to external fixation.

## Introduction

Distal radius fractures are common and produce a major orthopaedic workload. These injuries are sustained overwhelmingly from low energy falls, usually from a standing height by an increasingly osteoporotic population [1]. In a recent study, patients treated for a distal radius fracture in South East Scotland had an average age of 55.5 years [1]. This group of patients have high functional demands and are often still in active employment. Treating the growing number of these difficult injuries presents a particular challenge for orthopaedic surgeons.

Fixed angle locking plates are widely used for the management of osteoporotic fractures. The use of locked volar plates for distal radius fractures is increasingly popular although there is little in vivo data to suggest superiority over other techniques. Proposed advantages of locked volar plating include improved pull out strength even in osteoporotic bone [2] and a volar surgical approach that avoids the need for an extensive dorsal dissection. The plate is positioned in a well padded area beneath pronator quadratus to avoid flexor tendon irritation and it is thought that patients tolerate volar wrist scars better than dorsal ones [3,4].

Surgeon and patient assessment of outcome do not necessarily correlate. When evaluating the success, or otherwise of surgery, several tools have been developed and validated to measure and report outcomes from the perspective of the patient [5]. We describe our experience and patient reported outcomes for locked volar plating of complex distal radius fractures.

## Patients and Methods

This study was reviewed and approved by our regional ethical review committee. Over a 12 month period, we treated 21 patients with complex type C distal radius fractures using locked volar plating. Plating was undertaken by or under the supervision of one of two consultant orthopaedic surgeons with a specialist interest in upper limb surgery. Patients who were unfit for surgery, unable to give informed consent or who had low functional demands were not included. Fracture classification was performed preoperatively and confirmed at the time of surgery using the AO classification system [6]. Post-operative assessment involved a wound check at 2 weeks with routine radiographic imaging, a further appointment at 6 weeks at which point formal referral for physiotherapy was made and another outpatient visit at 3 months. Patients were invited for a further clinical assessment at 6 months and all of the patients accepted this offer.

A final assessment was performed at a minimum of one year post operatively and this included radiographic, clinical and functional measures including range of movement, grip strength, pain scores, the patient rated wrist evaluation (PRWE) score [7,8] and questions to directly assess patient satisfaction. The patient rated wrist evaluation score is derived from a patient completed questionnaire comprising two parts weighted equally for wrist related pain and function. It has been validated as a sensitive measure of recovery after distal radius fracture [7-10]. The score is ranged between 0 and 100 with higher scores representing less satisfactory outcomes. Fracture union was defined by the absence of local tenderness at the fracture site combined with radiographic evidence of trabeculae spanning the fracture site. Patients were asked to mark their level of pain on a 10 cm visual analogue scale, 0 representing "no pain" and 10 representing "the worst pain ever". Patients were also asked to complete a satisfaction questionnaire devised in our unit in order to grade levels of satisfaction relating to their treatment and recovery.

The questions asked were

1. How satisfied are you with the results of your surgery?

2. How satisfied are you with the relief of pain?

3. How satisfied are you with your ability to perform every day activities?

4. Would you have the same operation again?

5. Would you recommend this surgery to a friend?

### Surgical Technique

Surgery was performed under general anaesthesia. A proximal arm tourniquet was routinely used and prophylactic antibiotics administered before inflation. The surgical approach was through the sheath of the flexor carpi radialis tendon. The Synthes oblique 3.5 mm LCP T-plate was used for 17 patients and the 2.4 mm LCP distal radius plate (Synthes, Paoli, Pensylvania) was used for the remaining 4 patients. The plate was applied to the volar aspect of the distal radius under direct vision and fixed proximally using the oblong hole to allow fine adjustment, the fracture was reduced and temporary fixation was maintained with K-wires. The reduction and plate position were routinely checked under image intensification. Distal locking screws were subsequently sited so as to reach but not penetrate the dorsal cortex. A measurement of 2 millimetres was routinely subtracted from the distal screw length measurement in order to avoid penetration of the dorsal cortex and to minimise the potential for extensor tendon irritation. Distal locking screws were positioned aiming to site them 2 mm below the joint line in order to provide subchondral support [11]. A final check was made for plate and screw positions with image intensification using a standard postero-anterior view, two oblique views and a true lateral view of the wrist in order to ensure that the joint had not been penetrated [12].

None of the patients required bone grafting or bone substitute. Patients were followed up in the outpatient setting until clinical and radiographic union was achieved. Patients were routinely referred for physiotherapy. A further clinical assessment was made at 6 months and subsequent follow up at a minimum of 1 year with a patient satisfaction survey and patient rated wrist evaluation scoring. Grip strength was measured using a calibrated Jamar hydraulic hand dynamometer (Irvington, New York) and compared with the contralateral wrist as well as previously established normative data for this population.

## Results

Twenty-one patients with type C fractures of the distal radius were treated with locked volar plates over this period. There were 8 men and 13 women with an average age of 48 years (range, 22-67). The mean time to surgery was 4 days (range, 1-12) and a consultant surgeon with an upper limb interest was the primary surgeon or assistant for each case. All of the patients were right hand dominant. There were 8 left wrist injuries. A fall from a standing height was by far the most common mechanism of injury, reported by 19 patients. One patient was involved in a road traffic accident and a second suffered a rugby injury. None of the injuries were open.

All of the patients had achieved clinical and radiographic union by 3 months. There was no requirement for reoperation. Table 1 summarises patient details, fracture classification as well as range of movement and radiographic alignment after union. Type C3 fractures predominated: 4 type C 1 fractures, 8 type C 2 fractures and 9 type C 3 fractures. At 6 months, range of wrist movement was already approaching that of the contralateral wrist although comparing matched pairs demonstrates that there was still a significant difference in range of postoperative palmar flexion for both wrists at this time (p < 0.0001, Wilcoxon matched-pairs signed ranks test). No such difference can be shown for wrist extension (p = 0.4332, Wilcoxon matched-pairs signed ranks test).

**Table 1:** Patient and injury characteristics, early post-operative assessment (6 months)

**No**.	Age/Sex	Fracture classification	Palmar Flexion Injured/Contralateral (°)	Palmar Extension Injured/Contralateral (°)	Implant
1	53/F	23C2	65/80	75/75	3.5 mm T-plate

2	43/M	23C2	45/70	60/85	3.5 mm T-plate

3	43/F	3C3	60/65	80/80	3.5 mm T-plate

4	40/M	23C3	55/65	50/65	3.5 mm T-plate

5	67/F	23C2	65/70	60/70	3.5 mm T-plate

6	49/F	23C3	70/70	75/75	3.5 mm T-plate

7	22/M	23C2	52/65	70/70	3.5 mm T-plate

8	42/M	23C3	60/60	65/75	3.5 mm T-plate

9	60/M	23C2	55/70	70/80	3.5 mm T-plate

10	52/F	23C2	75/80	85/70	3.5 mm T-plate

11	26/M	23C2	65/65	65/65	3.5 mm T-plate

12	52/F	23C1	60/70	60/75	3.5 mm T-plate

13	42/F	23C1	65/80	55/70	2.4 mm LCP

14	48/F	23C2	55/75	75/65	3.5 mm T-plate

15	58/F	23C3	50/70	80/70	2.4 mm LCP

16	56/F	23C2	65/70	70/65	2.4 mm LCP

17	48/F	23C1	60/65	76/70	3.5 mm T-plate

18	48/M	23C1	66/70	70/55	3.5 mm T-plate

19	48/F	23C3	75/75	70/65	2.4 mm LCP

20	53/M	23C3	55/70	76/65	3.5 mm T-plate

21	62/F	23C3	74/75	55/70	3.5 mm T-plate

Patients reported high levels of satisfaction at final assessment. This assessment was made at an average of 15 months (range, 12-21). 100% of patients reported "very high" or "high" levels of satisfaction with their surgery at final review but more specific questioning identified that 9.5% remained dissatisfied in some way with respect to residual pain or functional limitation. These data are presented in Table 2. Patients achieved a good recovery in grip strength compared with the contralateral wrist at 6 months. Table 3 shows that despite this recovery in grip strength, at 6 months there is still a statistically significant difference between injured and non-injured wrist grip strength (p = 0.0002, Wilcoxon matched-pairs signed-ranks test). There were no cases of extensor or flexor tendon rupture and no wound complications. Low visual analogue scores for pain indicate good symptomatic relief of pain and patients reported good functional patient rated wrist evaluation scores also. Low pain visual analogue scores corresponded well with low pain components of the patient rated wrist evaluation score (Table 4). One patient was carpally malaligned on final x-ray images but she reported good function and no further surgical intervention is planned.

**Table 2:** Patient satisfaction

	"Highly satisfied" or "Satisfied"	"Dissatisfied" or "Highly dissatisfied"
Q1 How satisfied are you with the results of your surgery?	21 (100%)	0 (0%)

Q2 How satisfied are you with any symptoms of pain in your wrist?	19 (90.5%)	2 (9.5%)

Q3 How satisfied are you with your ability to perform every day activities?	19 (90.5%)	2 (9.5%)

	**"Definitely yes" or "yes"**	**"No" or "Definitely not"**

Q4 Would you have the same operation again?	21 (100%)	0 (0%)

Q5 Would you recommend this surgery to a friend?	21 (100%)	0 (0%)

**Table 3:** Grip strength measurements

	Grip strength Injured Wrist (Kg)	Contralateral Grip strength (Kg)	Normative population grip strength for sex and age (Kg)	Grip strength as % of contralateral grip strength
1	24	26	24	92.3

2	35	44	48	79.5

3	34	35	29	97.1

4	44	50	48	88

5	24	26	25	92.3

6	27	28	25	96.4

7	41	45	45	91.1

8	45	44	42	102.3

9	45	45	46	100

10	20	24	26	83.3

11	38	46	44	82.6

12	25	25	26	100

13	28	24	29	116.7

14	22	20	26	110

15	20	26	27	76.9

16	24	28	27	85.7

17	26	30	26	86.7

18	40	40	42	100

19	24	26	26	92.3

20	35	40	42	87.5

21	26	28	26	92.9

**Table 4:** Self reported pain and Patient rated wrist evaluation (PRWE) scores

	Pain Visual analogue score (/10)	Pain component-PRWE Score (/50)	Total PRWE Score (/100)
1	2.2	13	23

2	3.7	13	42

3	1.5	5	7

4	0	0	5

5	0	0	7

6	2	5	9

7	5.6	20	50

8	0	0	16

9	0	4	9

10	3.1	11	29

11	2.4	2	14

12	3	11	25

13	0	0	0

14	0	0	4

15	0	0	10

16	2.4	13	37

17	0	0	17

18	6	7	23

19	2.1	5	27

20	3.9	11	37

21	0	0	0

## Discussion

Complex articular fractures of the distal radius represent an increasing challenge for surgeons and for the design of new surgical implants. The popularity of locked volar plating continues to grow however, previous reports of successful outcomes concentrate on radiographic and surgeon orientated measures of success. Several reports use the Gartland and Werley score to evaluate outcomes after distal radius fracture. Although widely used, this tool has not been validated and has been criticised heavily [13-15]. The patient rated wrist evaluation score has been shown to be much more sensitive to recovery after distal radius fracture than the two more commonly used assessment tools, the Disabilities of the Arm, Shoulder and Hand (DASH) score or the Gartland and Werley score [13]. There is extensive work to show that locked volar plates are well tolerated, allow early movement and maintain position even for intra-articular fractures [16,17]. There is debate as to the true benefit of locked volar plating over augmented external fixation, which remains the mainstay of treatment for complex articular injuries [16-19]. Patient satisfaction is a complex idea and incorporates success not just of the surgical procedure but also of the consent process and subsequent rehabilitation. It is difficult to measure but patient satisfaction questionnaires/surveys are frequently used [8,13,15].

The population in our study were around the same age as previously studied groups treated with locking volar plates [16,17] and slightly younger than the average age of patients sustaining this injury in South East Scotland [1]. Nevertheless, we recognise that patients are actively selected for this surgical intervention based on patient and fracture characteristics. The low energy required to sustain these fractures despite the relative youth of this patient group is a concern and may herald future difficulties for fracture care among an increasingly osteoporotic population.

Beaule et al identified pain as a key predictor of satisfaction among patients recovering from distal radius fracture [20]. Our data supports Beaule's contention; showing low levels of reported residual pain, low visual analogue scores for pain, low patient rated wrist evaluation scores matched high levels of patient satisfaction. No difference could be determined when pain scores, satisfaction levels or residual grip strength were compared between patients injuring the dominant wrist or the non-dominant side. Of the 21 patients at a minimum of 1 year follow up, 9 had no pain. Of the remaining 11 patients, 7 described mild pain (visual analogue score 0-3) and 4 described moderate pain (visual analogue score 4-7).

Previous work has shown that patients achieve most of their improvement in range of movement and grip strength by 6 months although they may continue to improve up to around 18 months [21,22]. We have published previous work to examine normative grip strength in the South East Scotland population and have demonstrated that bilateral grip strength is normally roughly equivalent [23]. All of our patients achieved a recovery to over 79% of contralateral grip strength by 6 months and most had achieved over 90% of contralateral grip strength by this time.

None of our patients suffered any extensor tendon or flexor pollicis longus rupture although we have previously noted these complications among other patients. Both of these complications are well described [24,25] and we believe care should be taken intra-operatively to ensure that the dorsal cortex is reached but not penetrated by the distal locking screws and the pronator quadratus is laid back over the metalwork, tacking it into place where possible. Both extensor tendon and flexor pollicis longus rupture have been reported late in the literature and should be vigilantly looked for [25,26]. Our patients are routinely followed up with physiotherapy and subsequently asked to return to clinic should they have any further problems. Final radiographic examination at union confirmed that the locked volar plate maintained satisfactory position in keeping with previous studies.

It is well established that locked volar plating for distal radius fractures performs well when assessed by surgeon oriented and technical measures of success. Our study confirms that this technique is useful for complex articular injuries and performs well when judged by patient reported outcomes and measures of satisfaction. Despite statistically detectable differences in post-operative palmar flexion and grip strength, patients reported low pain scores and high levels of satisfaction. Further work should address whether locked volar plating produces superior patient reported outcomes and satisfaction compared with external fixation.

## Competing interests

The authors declare that they have no competing interests.

## Authors' contributions

REA reviewed the patients and drafted the manuscript. LAKK reviewed the patients. REC and JMcE perfomed or supervised the surgery and reviewed the manuscript. All authors read and approved the final manuscript.
